# Otology

**Published:** 1904-08-06

**Authors:** 


### OTOLOGY.

**Suppurative Otitis Media.**—A. C. Bardes[1] has discussed this condition. The persistence of ear-discharge is due, in nearly all cases, to the neglected or imperfect treatment of acute otitis media. The nasal chambers in civilised man are seldom in a healthy state, and nearly all aural disease has its beginning in the nasal passages. The lymphatic connection between the nose and ear is so intimate in young children that even gastro-intestinal or respiratory trouble is usually associated with congestion of the middle ear; also, in young children, the Eustachian tubes are disproportionately shorter and more pervious than in adults; for these reasons a running ear is frequent in infants. The old idea that it is dangerous to stop a running-ear is based on the experience that the sudden stoppage of a discharge from interference with its escape gives rise to dangerous symptoms. The abolition of pus formation is always desirable, but the retention of pus never. Three persons in every hundred with running-ear die from its effects, and in others the discharge tends to shorten life. When purulent otitis persists, first the long process of the incus, and next the head of the malleus tend to become necrotic, but fortunately the stapes and inner wall of the tympanum rarely become necrotic, for which reason labyrinthine suppuration is uncommon. Multiple perforations of the drum-head generally indicate tubercular disease, and are incurable. Discharge with low-lying perforation is more amenable to treatment than when the perforation is high up. A hole in the upper posterior quadrant points nearly always to disease of the incus, and one in Shapnell's membrane points to disease of the malleus and incus. The latter perforation only impairs hearing slightly, for the middle ear proper is usually in such cases shut off from the attic, which alone suppurates. The discharge in running ears is either from an "unhealthy granular surface," in which case it is muco-purulent and odourless, or from diseased bone, when it is usually ill-smelling and irritating. In treatment the author finds astringent solutions of great use. It is best to begin with weak solutions, and gradually increase their strength. Those most recommended are a solution of nitrate of silver, 5 to 80 grains to the ounce; zinc chloride, 2 to 20 grains; carbolic acid, 10 to 120 grains; formalin, 5 to 40 grains; and tincture of iodine, 5 to 60 grains. Syringing, as carried out by the patients themselves, is not as a rule satisfactory, for fluid is left behind and acts badly. The best ear-drops for the patient to use at home are those of a 20-per-cent. alcoholic solution of boracic acid. Ossiculectomy, with curetting, is recommended before resorting to the radical mastoid operation.

**Mastoiditis.**—E. H. Pomeroy [2] has written on this subject. Though, as a rule, there is severe pain in the mastoid process, yet cases are met with, following acute otitis, in which there is advanced mastoiditis without other pain than the original earache, and some are met with even without any pain. The displacement downwards and forwards of the pinna is almost pathognomonic of the disease. Sometimes an incision through the periosteum over the mastoid will cure the affection. The point the author wishes especially to emphasise is that it is necessary to examine the tympanum in every case of serious illness in infants. The work of Ponfick is referred to in this connection. Pomeroy writes, "It may be safely stated that every infant dying under the age of one year has pus, or inflammatory processes established, in the middle ear." Hence paracentesis of the membrana tympani should be performed, which may be termed the prophylactic treatment of mastoiditis. If mastoiditis is present, the mastoid cells are to be removed. If surgical treatment is refused by the parents or not carried out for other reasons, then, to overcome bacterial infection sulphur should be given to saturation of the system therewith. For patients able to take a pill, calcium sulphide in doses of one-tenth grain to one-quarter grain in the pill form should be given three or four times daily. For infants one or two grains of the one per cent. powdered hepar-sulphur every two to four hours may be given.

**The Distance of the Antrum from the Cortex of the Mastoid Process.**—Phillip D. Kerrison,[3] in view of the diversity of opinion as to the maximum depth of the antrum, records some careful investigations he has made in the endeavour to obtain more definite data on the subject, and to formulate safe guiding rules for the surgeon who has to open that cavity. The following are the maximum depths of the antrum as estimated by different authorities: Gruber, 15 mm. (a little less than $\frac{5}{8}$ inch); Politzer, 15 mm.; Buck, $\frac{3}{4}$ inch; Deuch, $\frac{7}{8}$ inch; Schwartze, 25 mm. (about 1 inch); Broca, 29 mm., or about $1\frac{1}{6}$ inches. These variations are partly due to the measurements being made from different points on the cortex. All agree that the antrum may possibly be reached at a depth of 8 to 10 mm. ($\frac{3}{8}$ inch). The author points out that the antrum is best approached from the part of the mastoid cortex which lies nearest to it, and that this part is nearly always the supra-meatal triangle just behind the spine of Henle. He has found, in the specimens investigated, that the depth of the antrum is always less than the length of the postero-superior wall of the meatus, that in the great majority of bones it is not over 12 mm., and that it is never greater than 15 mm., or $\frac{5}{8}$ inch, and that therefore in operating it is not safe to go deeper than $\frac{5}{8}$ inch.

1 Med. Record, March 5, 1904, p. 369. 2 Ibid, March 12, 1904, p. 414. 3 Manhattan Eye and Ear Hospital Reports, March 1904, p. 90.

*(To be continued.)*

